# Spatial accessibility and equity of community healthcare: unraveling the impact of varying time and transport mode

**DOI:** 10.3389/fpubh.2024.1380884

**Published:** 2024-07-10

**Authors:** Jianhua Ni, Zhuo Wang, He Li, Jie Chen, Qi Long

**Affiliations:** ^1^Anhui Province Key Laboratory of Wetland Ecosystem Protection and Restoration, Anhui University, Hefei, China; ^2^School of Resources and Environmental Engineering, Anhui University, Hefei, China; ^3^Hefei Emergency Centre, Hefei, China; ^4^Department of Emergency Surgery, The Second Affiliated Hospital of Anhui Medical University, Hefei, China; ^5^School of Architecture and Surveying Engineering, Datong University, Datong, China; ^6^Anhui Mobile Communication Co., Ltd., Hefei, China

**Keywords:** spatial equity, spatial accessibility, community healthcare, varying time, multimode

## Abstract

**Background:**

Achieving a higher level of accessibility and equity to community healthcare services has become a major concern for health service delivery from the perspectives of health planners and policy makers in China.

**Methods:**

In this study, we introduced a comprehensive door-to-door (D2D) model, integrating it with the open OD API results for precise computation of accessibility to community hospitals over different transport modes. For the D2D public transit mode, we computed the temporal variation and standard deviation of accessibility at different times of the day. Additionally, accessibility values for D2D riding mode, D2D driving mode, and simple driving mode were also computed for comparison. Moreover, we introduced Lorenz curve and Gini index to assess the differences in equity of community healthcare across different times and transport modes.

**Results:**

The D2D public transit mode exhibits noticeable fluctuations in accessibility and equity based on the time of day. Accessibility and equity were notably influenced by traffic flow between 8 AM and 11 AM, while during the period from 12 PM to 10 PM, the open hours of community hospitals became a more significant determinant in Nanjing. The moments with the most equitable and inequitable overall spatial layouts were 10 AM and 10 PM, respectively. Among the four transport modes, the traditional simple driving mode exhibited the smallest equity index, with a Gini value of only 0.243. In contrast, the D2D riding mode, while widely preferred for accessing community healthcare services, had the highest Gini value, reaching 0.472.

**Conclusion:**

The proposed method combined the D2D model with the open OD API results is effective for accessibility computation of real transport modes. Spatial accessibility and equity of community healthcare experience significant fluctuations influenced by time variations. The transportation mode is also a significant factor affecting accessibility and equity level. These results are helpful to both planners and scholars that aim to build comprehensive spatial accessibility and equity models and optimize the location of public service facilities from the perspective of different temporal scales and a multi-mode transport system.

## 1 Introduction

Spatial accessibility and equity of healthcare services, which are becoming increasingly important for policymakers and health practitioners, play a crucial role in the health and well-being of residents. Convincing evidence has demonstrated that spatial barriers between demand and supply lead to low healthcare availability and decreased uptake of preventive services, which may result in poorer health levels (1). However, planners and scholars have been unable to comprehensively evaluate spatial equity as it has not been previously operationalized (2–6).

Spatial equity is vital in public facilities as it relies on the general principle that all residents should enjoy equal spatial proximity or spatial separation from the perspective of supply and demand (4, 7–9). Various analytical methods have been used to analyze the spatial equity of healthcare facilities. Accessibility is a critical metric for evaluating spatial equity. Accessibility usually refers to the ease with which a destination location (e.g., facilities at that location) can be reached from an origin location (e.g., people at that location) based on a transport network (10). The methods used to compute accessibility vary among studies. In literature, accessibility measures are mainly classified into two major categories: location-based accessibility and person-based accessibility (11–13). In general, location-based accessibility is defined as the degree to which transport networks enable people to reach their desired destination (14). Common measures of location-based accessibility include travel time or travel distances, the gravity model, and the two-step floating catchment area (2SFCA) method as well as a series of extended methods (15–20). Location-based measures, which only require rough and small amounts of aggregated data, have been widely applied in large areas. However, the main limitation of traditional location-based measures is that they usually employ static metrics to evaluate accessibility levels which may vary throughout the day (21, 22).

In fact, accessibility components (people, transport, activities) are most often dynamic in nature. Therefore, accessibility results ought to also fluctuate during the day based on people’s mobility, temporality of traffic flow, and the opening hours of facilities. The use of the time dimension as a critical factor has been widely recognized in literature with the development of person-based accessibility research. The majority of person-based measures are based on the time-geographic framework. Compared to location-based measures, they can better capture the nature of individual activity behaviors under different spatiotemporal constraints. However, such person-based measures are only used in personal accessibility research and are difficult to apply to large-scale macro research like location-based measures (12, 15) because of the privacy and high cost of obtaining large samples of personal data.

The rapid development of Location Big Data technologies has made it possible to obtain a large amount of spatiotemporal location data (23, 24). Although these spatiotemporal big data cannot meet the demand of person-based accessibility studies for high-precision data, they can provide a new data source for location-based accessibility research. In recent years, despite progress in time-dependent location-based accessibility modeling (25–27), only a few studies have considered the differences in spatial equity of accessibility mode at different times (28–30). Spatial equity based on static accessibility remains static and cannot reflect the results of possible fluctuations. Neglecting the dynamic nature of cities probably leads to biased or even misleading accessibility results and unreliable outcomes in terms of inequity.

Furthermore, the transport mode in urban areas is crucial for accessibility and equity research. Traditional accessibility measures assume that all people travel by car to seek healthcare services. However, in reality, several kinds of transport modes are available in big cities, resulting in great variation in the spatial accessibility and spatial equity computed using different transport modes (31–34). While numerous studies focus on the impact of different modes on spatial accessibility and the importance of integrating multiple modes to compute accessibility, far less attention has been paid to the ways in which equity of accessibility is influenced by different transport modes. This may in part be a corollary of the fact that the real travel time of accessibility of different transport modes is difficult to collect.

To summarize, existing studies often overlook the influence of the dynamics of temporality and multiple transport modes on spatial accessibility and equity in urban environments. To fill the current gap, this study computed the temporal variation and standard deviation of accessibility to community healthcare at different times of the day for the D2D public transit mode in Nanjing. For comparative analysis, accessibility values for three other different transportation modes were also computed. Additionally, this paper introduced Lorenz curve and Gini index to assess the differences in equity of community healthcare across different times and transportation modes. The primary objective of this study is to explore how the open hours of community hospitals, fluctuations in traffic flow, and various transport modes on spatial accessibility and equity analyses. The results are supportive for the spatiotemporal evaluation and optimization of public service facility location.

## 2 Materials and methods

### 2.1 Study area and data

**Figure 1:**
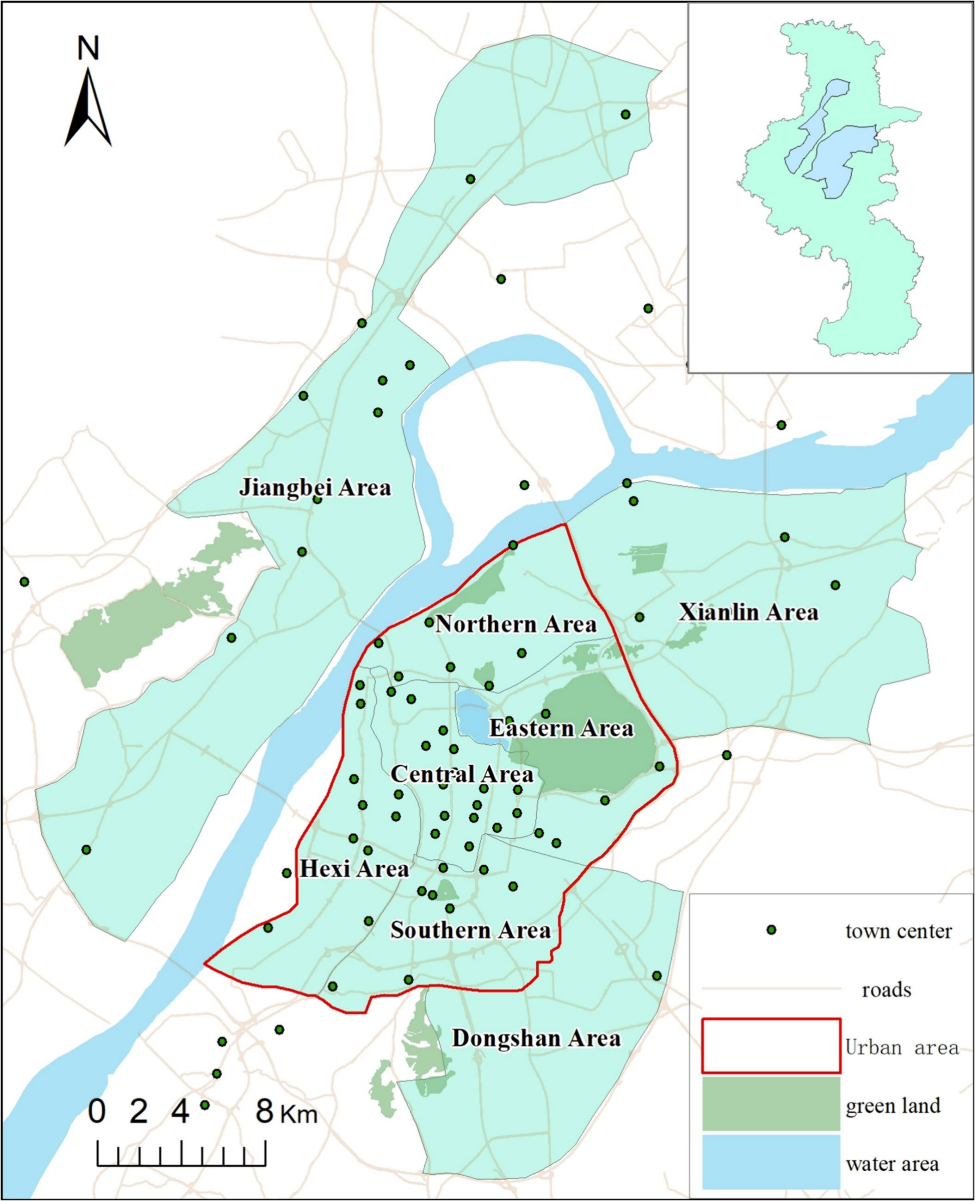
Study area.

The study area is in the city of Nanjing, the capital of the Jiangsu province, which is an important central city in eastern China (see Figure 1). The metropolitan area includes the main urban area and three sub-urban areas (Jiangbei, Xianlin, and Dongshan). The main urban area is located to the east of the Yangtze River with ring road to the west, comprising the Central, Hexi, Southern, Eastern, and Northern Areas.

In 2021, the total population of the study area was 4.87 million where male and female comprises 51.05% and 48.95% population, respectively. The population aged 0–14 years comprises 12.59% of the total population, while the proportion of the older adult aged 60 and above is 19.36%. Compared with other provincial capitals, Nanjing’s population has higher educational attainment and income levels. According to the 2021 Statistical Yearbook of Nanjing, the average *per capita* Gross Regional Product (GRP) was $24,217, ranked the first in China. And Nanjing hosts a total of 51 colleges, with the proportion of the population having attained an education level of college or higher being 35.23%, ranked the second in China.

In Nanjing, residents have access to various transportation modes to reach hospitals. According to the statistics, six of them are the most popular modes, which account for more than 98 percent (35). The six transportation modes are: walking (W), riding by bicycle (B), driving (D), public transit (PT), riding by e-bicycle (EB), and riding by motorcycle (MC). The number of e-bicycles and car amount were approximately 4.83 million and 1.46 million units respectively, with the former being the primary mode of transportation in Nanjing. Based on our previous survey, it was observed that when individuals seek healthcare services at hospitals, approximately 25% of them opt for public transportation, while over 20% of respondents choose e-bicycle riding as their mode of transportation (34).

#### 2.1.1 Healthcare service facility

**Figure 2:**
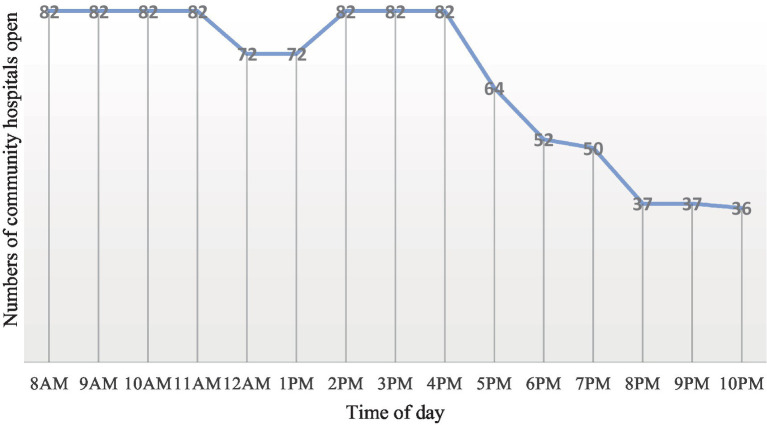
Number of community hospitals open during the day.

Community healthcare holds significant importance in the development of Chinese society, and its level of development serves as a reflection of the aspects of social equity and welfare. Community hospitals are a type of healthcare facility capable of providing fundamental medical services. Each community in Chinese cities has one own dedicated community hospital. To address the strain on large hospitals, the Chinese government is vigorously promoting the development of community hospitals. Therefore, we choose community hospitals as the study object in this study. The information on the operating hours and addresses of community hospitals was obtained entirely from the website (36). According to statistics, the majority of urban residents seek health services at community hospitals from 8 AM to 10 PM. Therefore, the temporal scope for spatial accessibility and equity in this paper was confined to this timeframe, and data was collected on an hourly basis. The open number of these community hospitals are shown in Figure 2 based on statistics.

**Figure 3:**
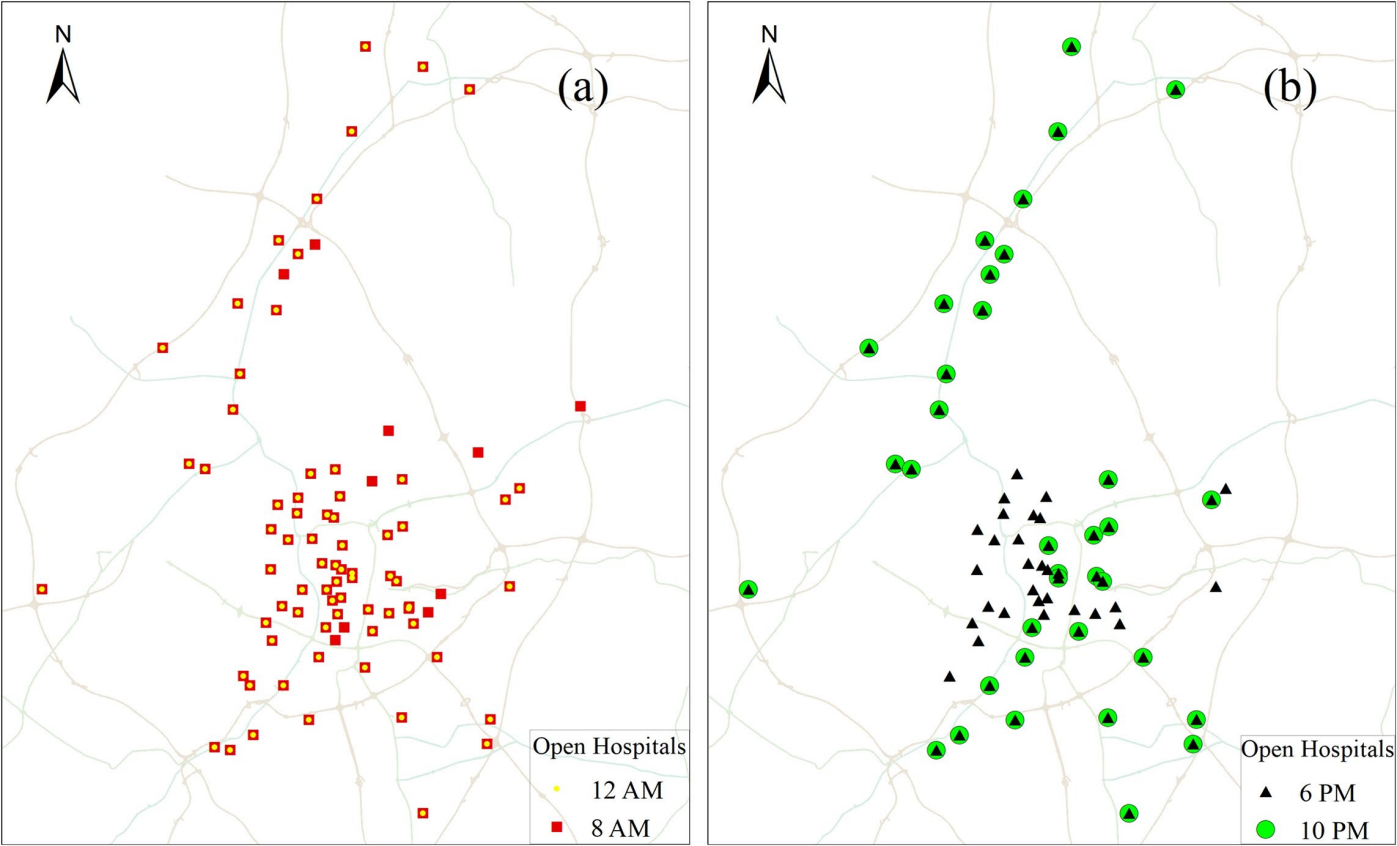
Spatial distribution of community hospitals that are open at four different times: **(a)** 8 AM and 12 PM; **(b)** 6 PM and 10 PM.

The spatial distribution of community hospitals in the study area is illustrated in Figure 3A at 8 AM and 12 PM, while Figure 3B displays their distribution at 6 PM and 10 PM. To mitigate the impact of classical edge effects inherent in spatial analysis, community hospitals within a 2 km radius of the study area are also considered as part of the study.

#### 2.1.2 Population multilevel cell division of the study area

**Figure 4:**
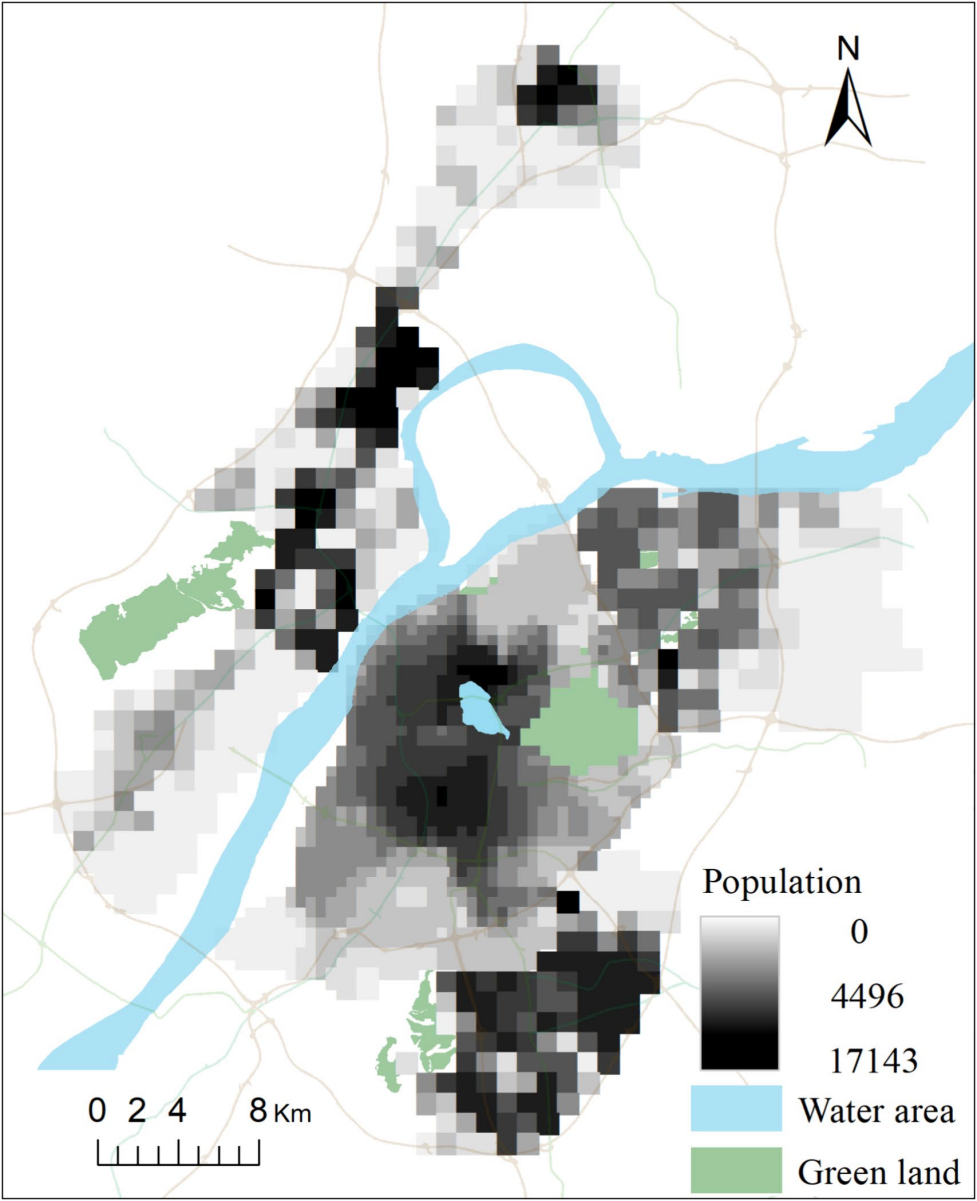
Grid-based spatial distribution of population in study area.

When computing accessibility, previous literature extracted population data from national census records, which often take the whole administrative region as a single discrete point in space. However, the spatial resolution of this kind of data is too low to meet the needs of high-precision accessibility computing. In this study, we distributed population data among grid cells, rather than concentrating it at a single point in the administrative centre (Figure 4). The population data was downloaded from the Resource and Environment Science and Data Centre. To account for varying population densities and reduce the computational load, we implemented two distinct grid cell sizes. Specifically, a grid size of 500 m × 500 m was utilized for the main urban area, while the sub-urban areas were represented using a grid size of 1,000 m × 1,000 m. The study area consisted of a total of 1,512 cells, with 964 located in the main urban area and 548 in the suburban areas.

### 2.2 Methods and definitions

#### 2.2.1 Travel time to the closest hospital based on the D2D method

Although various types of accessibility models have been proposed in healthcare field, the minimum travel time stands out as one of the most direct and effective method for accessibility evaluation. This method is particularly well-suited for research focused on seeking services nearby. Therefore, we specifically applied the minimum travel time method from each population point to the nearest community hospital.

Traditional methods for computing travel time often rely on simple, static road network models, which can deviate significantly from actual travel time outcomes. Results obtained from open map APIs, on the other hand, are grounded in real road network structures and dynamic traffic cost statistics, rendering them more accurate than those derived from traditional methods. Despite this accuracy, factors such as additional walking, parking costs, and time expenses for uncollected roads outside the road network can impact final accessibility results. To address these considerations, this paper introduces a D2D model, integrating it with crawled OD cost API results to enhance the precision of accessibility computations at different times.

**Figure 5:**
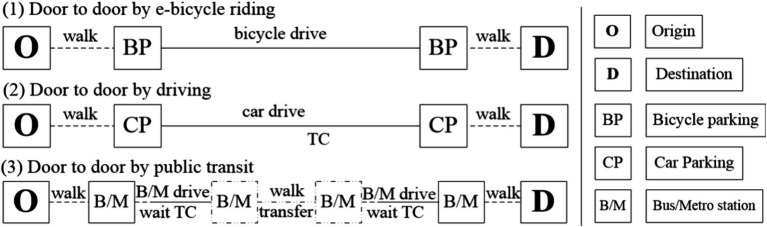
Three types of D2D transport modes.

In contrast to traditional methods, the D2D method takes into account more detailed travel aspects, including traffic congestion, necessary transfer times, and walking durations, all of which can significantly influence travel cost results (37). Based on the survey conducted in the study area in our previous study, the most frequently used transport modes to the health service facilities mainly include e-bicycle riding, driving, and public transit (34). To show the entire parts of the journey, we introduce three D2D models: riding, driving, and public transit, as illustrated in Figure 5.

The total travel times for the three types of transport modes using the above D2D method are expressed in Eqs. (1)−(3):

(1) $$T_{R}=T_{OB}^{walk}+T_{BB}^{riding}+T_{BD}^{walk}$$

(2) $$T_{D}=T_{OP}^{walk}+T_{PP}^{driving}+T_{PD}^{walk}$$

(3) $$\begin{array}{l} T_{PT}=T_{OB\left(orOM\right)}^{walk}+T_{BB\left(orMM\right)}^{Bus\left(orMetro\right)}+\\ \left(\;T_{BB\left(orMM\right)}^{Bus\left(orMetro\right)}+T_{TC}+T_{TT}+\right)\\ T_{WAIT}+T_{BD\left(orMD\right)}^{walk} \end{array}$$

where $T_{OB}$ , $T_{OP},$ and $T_{OM}$ are the necessary walking times from the origin to the bus station, metro station, and car parking, respectively. $T_{MM}$ , $T_{BB}$ , and $T_{PP}$ are the travel time by metro, bus, and car, respectively. $T_{MD},T_{BD}$ , and $T_{PD}$ are the travel time from the metro station, bus stop, and car parking to the final destination, respectively. Optional travel time are denoted with ‘()’, encompassing $T_{BB}$ (possibly multiple times), $T_{TC}$ (traffic congestion), and $\;T_{TT}$ (traffic transfer). $T_{TT}$ is the transferring time for both bus/metro and stop/station. $T_{WAIT}$ is the waiting time for bus/metro arrival and departure.

**Figure 6:**
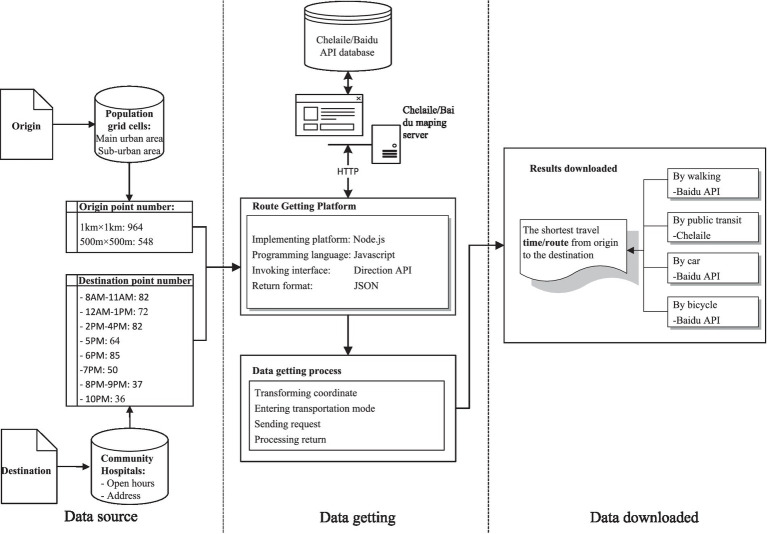
Technical flow chart of getting OD travel time of a given route.

The total travel time for a given route is defined as the shortest time from the origin to the destination among all possible paths for a specific mode. In our study, we designated the population cell centroids (refer to Figure 4) as the origin points and community hospital points (refer to Figure 3) as the destination points. While the Baidu Map API platform can provide dynamic travel costs for driving mode at different times of the day, it does not offer dynamic travel costs for public transit mode. Simultaneously, the variations in travel costs for riding modes are minimal in the study area. As a result, we collected travel times for driving and riding modes by integrating the D2D model with Baidu Map API data. Additionally, travel times for public transit mode were collected by integrating the D2D model with Chelaile API data. Chelaile platform can supply the dynamic travel costs in public transit mode for different time of the day. The computing procedures of the OD travel cost using Baidu Map APIs are shown in Figure 6.

In the computation of travel costs for the three D2D transport modes, the following rules were applied: In the presence of an off-road origin or destination point, we treat the transport mode traveling from this point to the nearest road as walking mode, with a set speed of 1.2 m/s. The parking time and searching time for e-bicycles are both set at 1 min, while for cars, they are set at 5 min. Additionally, the average waiting time for buses or metro is set at 3 min for each trip.

#### 2.2.2 Lorenz curve and accessibility Gini index

The above travel time is a highly used measure of the accessibility of any origin point to a public health service facility. However, this index fails to consider crucial information such as population demand, making it difficult to conduct intuitive and quantitative analyses of the inequality in the accessibility of public facilities. Delbosc and Currie (38), and Bhandari et al. (39) introduced an innovative methodology that leverages the Lorenz curve, a concept borrowed from economics, to evaluate the equity in the distribution of public transportation services. This approach was applied to analyze how public transportation resources are allocated relative to population and employment sectors in Melbourne and Delhi, providing a novel perspective on the equity of public transportation provision.

Additionally, the Gini coefficient is a quantitative index derived from the Lorenz curve, which can measure the overall degree of inequality. Jang et al. (33) employed a comprehensive approach utilizing the Lorenz curve and Gini coefficient based on accessibility to assess the spatial equity of public transportation in Seoul. There, in our study, we introduced the Lorenz curve and Gini index to assess how the general spatial equity of community hospital changes throughout the day. A higher Gini index indicates greater inequality, where individuals with high accessibility receive a much larger percentage of the total accessibility of the population. The Gini index values range from zero to one, where zero signifies absolute spatial equity, and one denotes perfect spatial inequity. Gini values under 0.2 indicate low spatial inequity, those between 0.2 and 0.5 denote medium spatial inequity, and values above 0.5 represent high spatial inequity. The mathematical formula of the Gini index is provided in Eq. (4).

(4) $$Gini=1-\sum_{k=0}^{n}\left(X_{k}-X_{k-1}\right)\left(Y_{k}-Y_{k-1}\right)$$

where $X_{k}$ is the cumulative proportion of the accessibility value with $X_{0}$ = 0, $X_{1}$ = 1, and Y is the cumulative proportion of the population value with $Y_{0}$ = 0, $Y_{1}$ = 1 ( k = 0 … *n*).

In this study, the acquisition and processing of travel time data, as well as the computation of the Lorenz curve and Gini index, were all implemented using the Python programming language.

## 3 Results

### 3.1 Temporal variation of accessibility by public transit at different times of the day

In a large Chinese city, people usually seek medical services at health facilities either by public transit or e-bicycles (34). Here, we use the public transit mode as an example to illustrate the impact of varying time on the spatial accessibility of community hospitals. According to statistics from the Baidu Map Transportation Big Data Platform (40), peak hours typically occur at 8 AM and 6 PM on weekdays, while traffic is relatively smooth at 12 AM and 10 PM during the day and night, respectively. Additionally, these four times coincide with significant fluctuations in the opening numbers of community hospitals. Therefore, we measured travel times between the centroids of the grid cell and community hospitals at 8 AM, 12 PM, 6 PM, and 10 PM.

**Figure 7:**
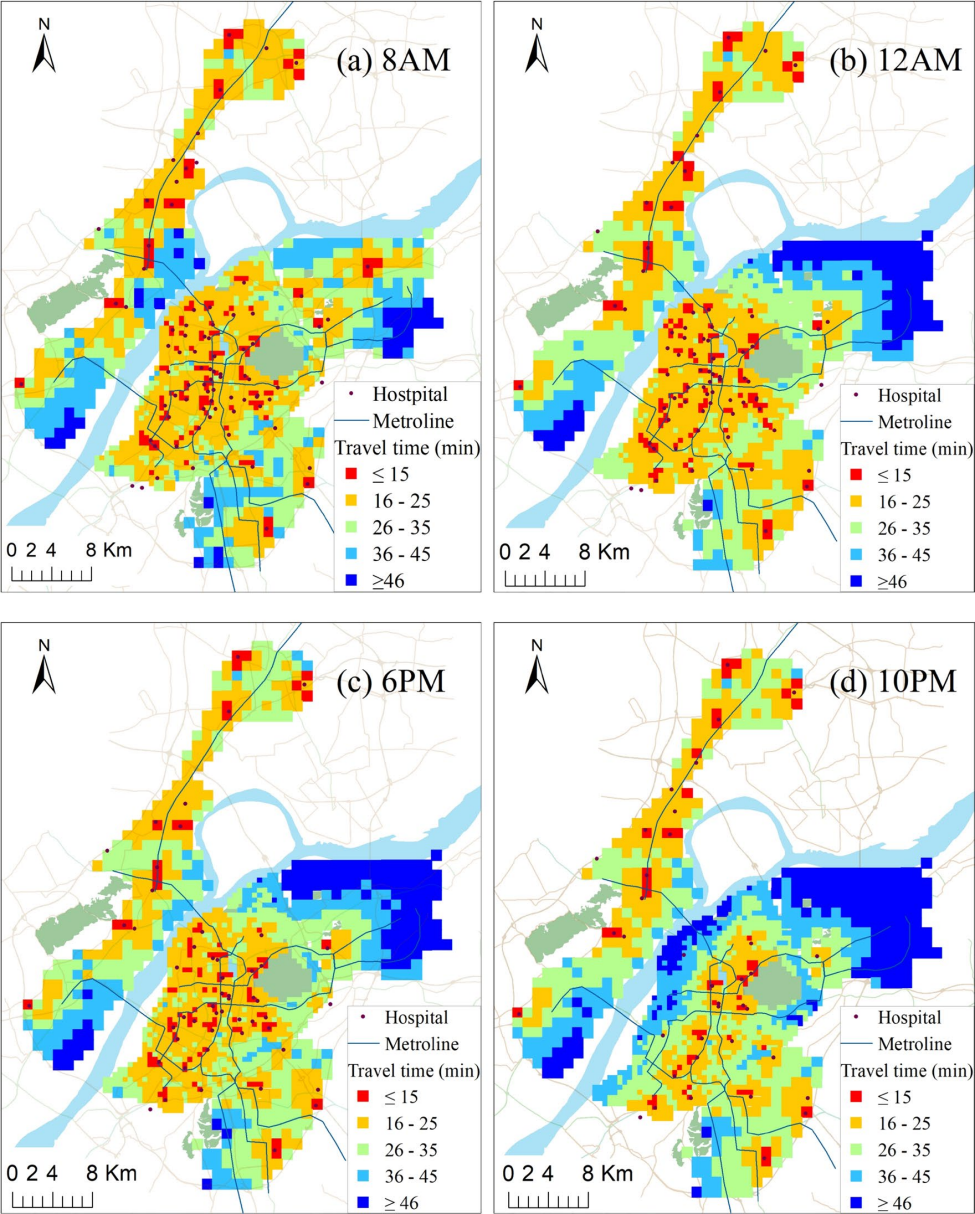
Travel time to closest facility of each population grid at four different times of day: (a) 8AM; (b) 12AM; (c) 6 PM; (d) 10PM.

The temporal variation in accessibility to the closest community hospitals is shown in Figure 7. In this study, we assume that smaller travel times indicate better accessibility, while larger travel times signify lower accessibility. As illustrated in Figure 7, four distinct accessibility patterns emerge, highlighting the significant impact of varying time on the accessibility of community hospitals.

In general, accessibility in urban areas is significantly higher at 8 AM, 12 PM, and 6 PM compared to suburban areas. Community hospitals are relatively concentrated in urban areas, and the spatial distribution of open community hospitals during these three periods is relatively even. However, overall accessibility is suboptimal. Only 10% of the population can reach their closest community hospitals within 15 min, the threshold set by the Nanjing Municipal Government for the service circle. Furthermore, although more community hospitals are open at 8 AM compared to 12 PM, the overall accessibility is worse at 8 AM. This is primarily due to the morning rush hour on weekdays, which is the most congested time of the day in the study area.

At 10 PM, a significant number of community hospitals are closed along Ruijin Road and in the Northern and Western Areas of urban areas, leading to a rapid increase in travel time for these regions, especially in the Northwest region of the main urban area. According to statistics, 45% of the population in the main urban area experiences an accessibility value exceeding 30 min at 10 PM. However, at 12 PM, only below 1% of the population faces a travel time exceeding 30 min. This suggests that, due to the relatively high population density of the main urban area, the closing time of many community hospitals has a more pronounced impact on accessibility and equity.

The majority of low-accessibility areas are situated in the suburban areas outside the urban centre. The number of open community hospitals in Dongshan remains relatively stable at all four moments, and accessibilities also tend to be consistent. However, in the other two suburban areas, their accessibilities are significantly influenced by the opening hours of community hospitals and traffic flow. For instance, traffic flow plays a crucial role in the accessibility of community hospitals in Jiangbei. Although the number open at 12 AM is 2 less than that at 8 AM, the accessibility of the central area is noticeably higher than that at 8 AM (compare Figures 7A,B), with a large population facing travel times above 36 min (indicated by blue color in Figure 7A). Xinlin area is vast, but there are very few community hospitals. Unfortunately, only 1 or 2 hospitals are open at 12 AM, 6 PM, and 10 PM, resulting in extremely low accessibility outside the community hospitals. More than 95% of the population has to spend more than 15 min to reach the closest community hospital located at the border with the central city for healthcare services. This situation is undeniably unfair, especially for low-income families without cars.

**Figure 8:**
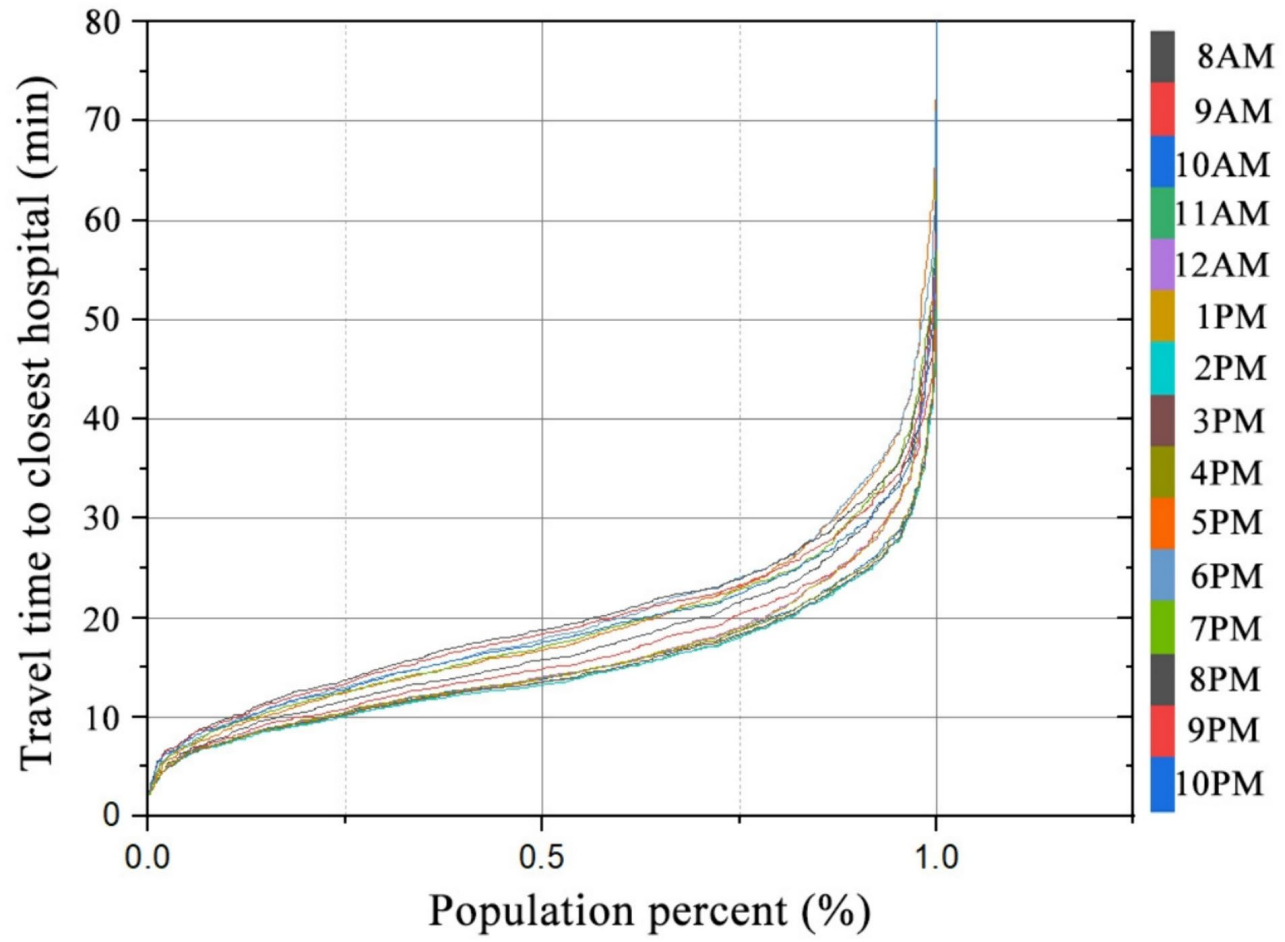
Relationship between percent of population and travel time to the closest hospital at different times.

Figure 8 illustrates the hourly relationship between the cumulative percent of the population and the temporal variation of travel time to the closest hospital from 8 AM to 10 PM. Approximately 50% of people in this study could reach the closest hospital within 20 min for all 15 time scenarios. However, while it took 18.8 min at 8 PM, it only took 13.2 min at 2 PM. The same number of hospitals are open between 8 AM and 11 AM, but the travel cost gradually decreases due to the impact of traffic mitigation. The curves for 12 PM and 1 PM almost completely coincide. The travel times at 2 PM, 3 PM, and 4 PM are similar to those at 8 AM and 9 AM but with a slight increase. At 5 PM, 6 PM, and 7 PM, the travel cost increases dramatically compared to that of 4 PM. The number of open hospitals at 8 PM, 9 PM, and 10 PM is almost the same. With the improvement of traffic, the travel cost gradually reduces. The results are consistent with the above conclusion that temporal accessibility changes significantly when considering the open hours of hospitals and traffic flow at different times.

**Figure 9:**
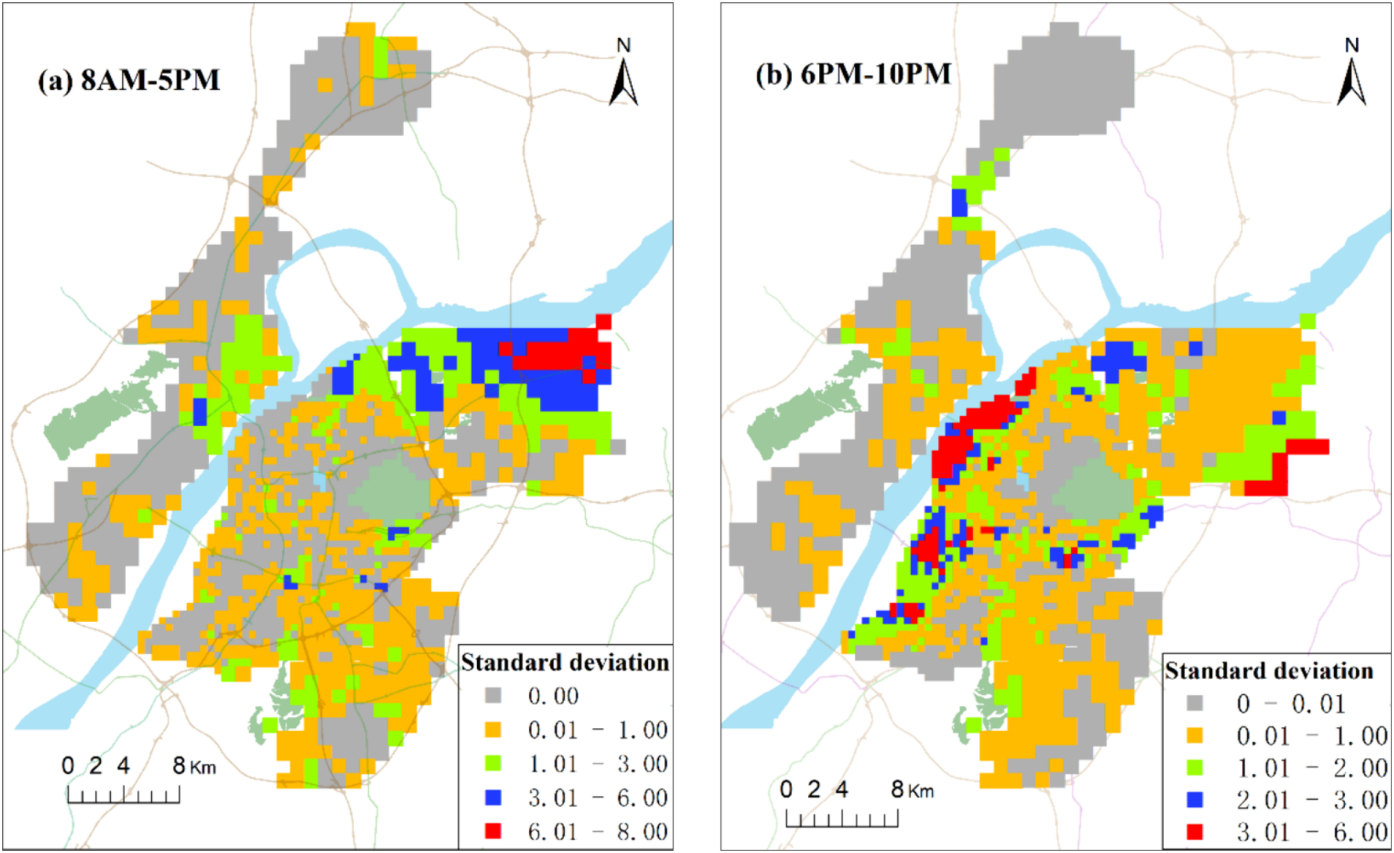
Spatial distribution of accessibility standard deviation of community hospitals in day **(a)** and night **(b)**.

To facilitate a better comparison of the spatial difference in accessibility dispersion between daytime (8 AM–5 PM) and nighttime (6 PM–10 PM), we computed the standard deviation based on accessibility at 10 different time periods during the day and 5 different time periods in the evening, as illustrated in Figure 9. The figure highlights the noticeable differences in accessibility standard deviation between day and night. The highest values of accessibility standard deviation are primarily concentrated in the northeast of Xianlin, exceeding 6.0. Additionally, during the daytime, the accessibility standard deviation is prominent along the Yangtze River area of Jiangbei, the northwest of Xianlin, and the northern part of the main urban area. In the evening, the maximum standard deviation of accessibility is mainly concentrated along the Yangtze River in the main urban area, although it is smaller than during the daytime.

In this context, it can be inferred that both the traffic congestion and open hours of community hospitals over time have an important impact on the accessibility of community hospitals, the latter (‘open hours’) more strongly. Whether the hospital is open or not directly determines the presence or absence of hospital services, while traffic congestion only affects the longevity of the travel time. The people in the main urban area along the river and the northwest of Xianlin are located on the borders of townships and have fewer opportunities to seek medical care as compared with residents of other areas. Therefore, extending the open hours of hospitals or adopting a dynamic allocation of healthcare service resources can possibly allow residents to enjoy medical services equitably.

### 3.2 Temporal variation of spatial equity at different times of the day

**Figure 10:**
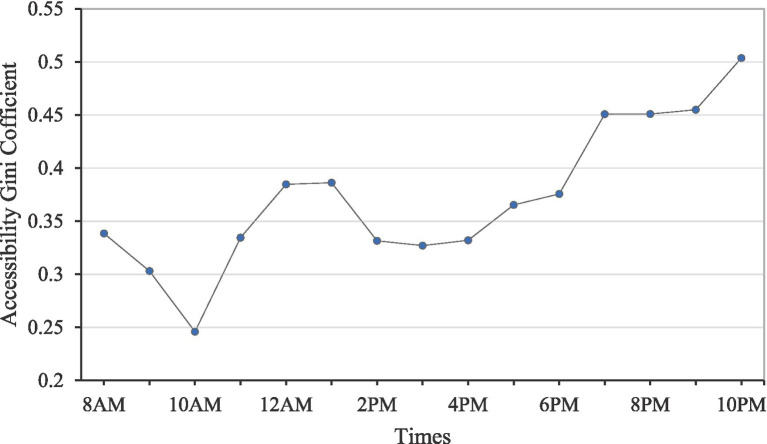
Varying curve of accessibility Gini value from 8 AM to 10 PM.

Figure 10 illustrates the variation curve of the accessibility Gini value from 8 AM to 10 PM. The number of open community hospitals remains constant from 8 AM to 11 AM. The time-varying trend of the Gini value between 8 AM and 11 AM generally aligns with that of traffic flow. According to the Baidu Map Transportation Big Data Platform, the morning peak around 8 AM is consistently the heaviest moment of the day in Nanjing. Correspondingly, the Gini value at this moment is the largest of the three time scenarios. As traffic conditions gradually ease, the Gini value steadily decreases after 8 AM. The Gini value reaches a turning point at 10 AM, exhibiting a ‘V’-shaped curve from 8 AM to 1 AM. By 10 AM, the Gini value falls to 0.246, marking the lowest point for the day. It can be concluded that 10 AM is the moment with the best overall spatial layouts in the study area.

The Gini value begins to gradually increase due to the onset of the noon rush hour. Affected by the reduced number of open community hospitals, the Gini values at 12 PM and 1 PM increase further than that at 11 AM (see Figure 2). At 2 PM, 3 PM, and 4 PM, several community hospitals reopen, resulting in smaller and stable Gini values during these periods. From 5 PM to 10 PM, the number of open community hospitals decreases, and the Gini value during these moments shows a gradually increasing trend. Until 10 PM, a significant number of daytime community hospitals along the Yangtze River in the main urban area and Xianlin suburban area, coupled with the high-density population in the main urban area, contribute to a rapid increase in the travel time from population points to the closest hospital. The Gini value is highest at 10 PM, reaching 0.504. Therefore, 10 PM is the moment with the worst overall spatial layouts in the study area.

### 3.3 Impact of different transport modes on spatial equity

**Figure 11:**
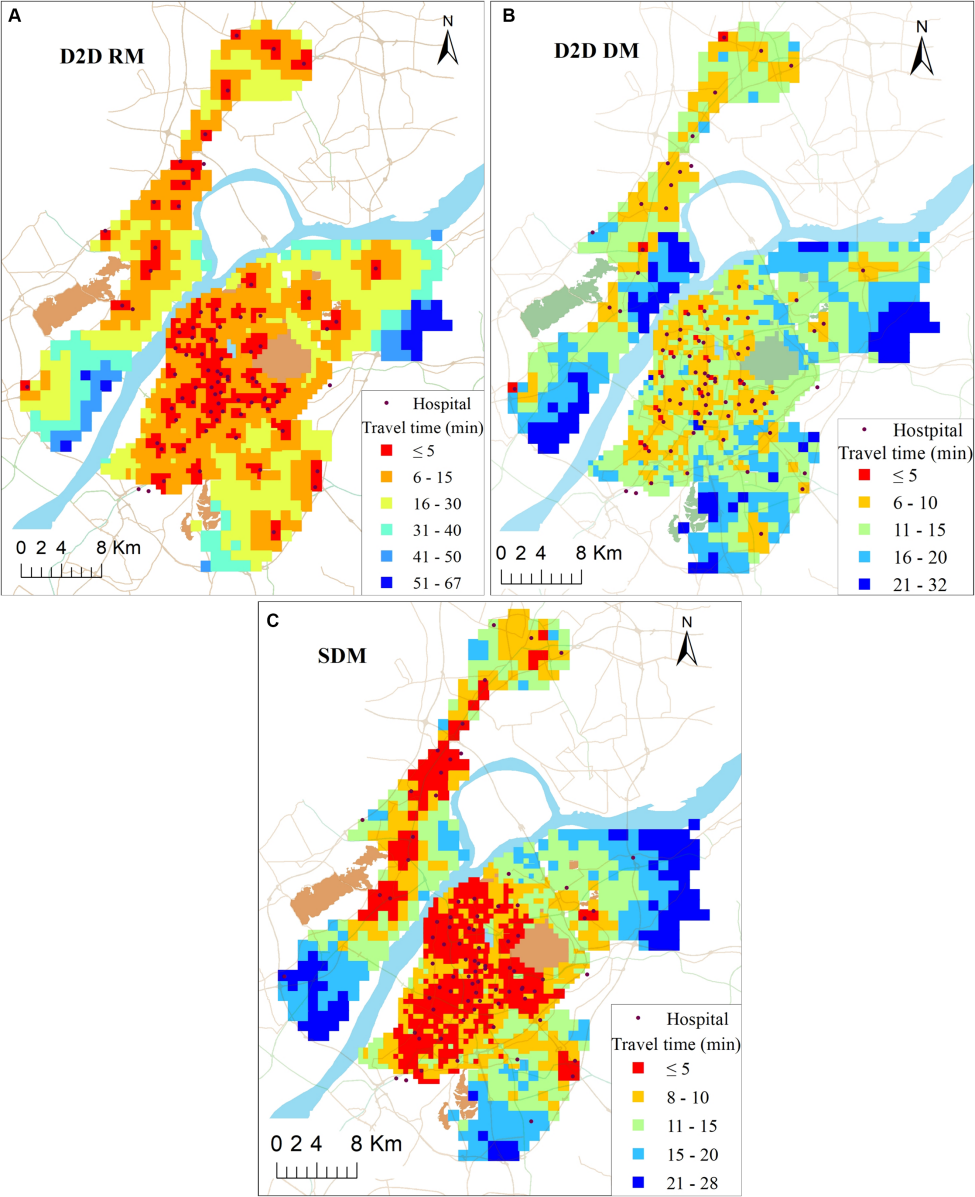
Travel time to closest facility at three transport modes (unit: minutes).

To compare the accessibility differences between the D2D public transit mode (PTM) and the other three modes, Figure 11 illustrates their accessibilities to the closest community hospitals, generated by the simple driving mode without the D2D method (SDM), D2D driving mode (DM), and D2D riding mode (RM), respectively. The simple driving mode is a commonly used transport mode in traditional accessibility studies, utilizing the speed limit value of the road or setting a fixed speed based on the road level. It does not consider every part of the OD travel route. The travel time of the SDM in this study is computed using the ArcGIS OD Cost Matrix Tool. The other three modes are all based on the above D2D model and Baidu Map API data. To mitigate the impact of traffic flow on spatial equity between different modes, the collected time of travel cost is set at 10 AM, which is the peak traffic period in the study area.

As shown in Figure 11, these three modes generate similar accessibility distribution patterns to the above PTM, with high accessibility values in the main urban area and low accessibility values in the suburban area. Additionally, proximity to the community hospital corresponds to better accessibility. However, each transportation mode exhibits unique spatial accessibility distribution characteristics.

Whether in the main urban area or suburban area, the accessibility of D2D driving mode is generally better than the public transit mode. In the main urban area, only 6% of the population have to spend more than 15 min to reach the closest community hospitals, while in the suburban area, 33% of the population face a travel time exceeding 15 min to reach the closest community hospitals. However, in D2D driving mode, people face challenges such as high parking costs and traffic congestion. In D2D riding mode, 40% of the total population can reach the closest community hospital within 5 min, which is notably better than other modes and areas. Compared with the other three transport modes, SDM shows better accessibility than the other three modes. The primary reason is that this mode ignores D2D details, significantly reducing travel costs.

**Figure 12:**
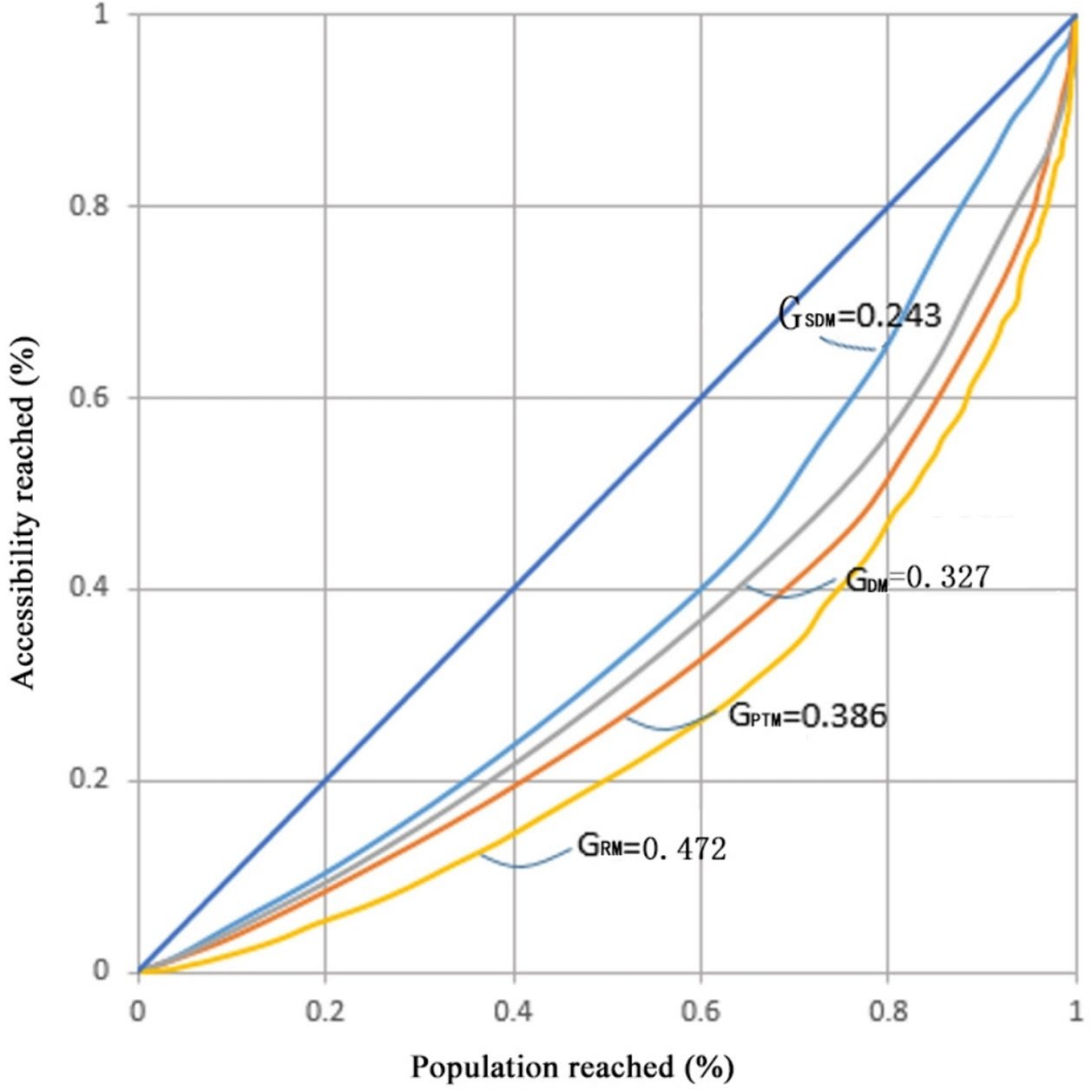
Accessibility Lorenz curve and accessibility Gini value of community hospitals under the four transport modes.

Taking into account the spatial accessibility of the four transport modes (DM, SDM, PTM, and RM), our attention is directed toward assessing the influence of these modes on the spatial equity of accessibility simultaneously. In order to depict the impact of different transport modes on the overall spatial equity of community hospitals, Figure 12 illustrates the accessibility Lorenz curves and the corresponding Gini values for each of the four transport modes.

As shown in Figure 12, the Lorenz curve and Gini values for the accessibility of community hospitals across the four transport modes exhibit considerable variations, underscoring significant differences in the spatial equity of community hospitals contingent upon the chosen transport mode. The Gini values of DM and PTM are at 0.327 and 0.386, respectively, representing levels deemed ‘relatively reasonable.’ This observation may be attributed to the comparatively faster driving speeds associated with DM and PTM, resulting in relatively smaller disparities in accessibility.

It is noteworthy that, despite the RM having a smaller accessibility standard deviation than the PTM in the study area, its Gini value is 0.086 higher than that of the PTM, reaching 0.472 and representing a ‘poor’ equilibrium level. This disparity may stem from the more pronounced polarization of travel time in the RM. Individuals in proximity to the hospital can reach it quickly, while those situated farther away must expend significantly more time to access healthcare services while riding. In the PTM, there are fewer instances of individuals experiencing extremely high or extremely low travel times. Therefore, it can be concluded that the spatial distribution of community hospitals in the PTM is more equitable compared to riding.

Compared with the other three transport modes, the Lorenz curve of the traditional SDM is closer to the line of absolute equity. The Gini value is only 0.243, which is the smallest among the four transport modes. Due to its ease of implementation in GIS, computing methods with SDM were frequently utilized in previous studies. However, this method overlooks traffic congestion and necessary walking, leading to an underestimation of the level of spatial equity of community hospitals. With the introduction of the D2D model and the reduction in OD opening API data collection costs, this mode is likely to be less used due to its tendency to overestimate the accessibility of healthcare services.

## 4 Discussion

Current research predominantly utilizes static metrics to gauge equity in healthcare accessibility. These studies frequently overlook the dynamic nature in accessibility and equity over space and time, which are inherent to the constantly evolving landscape of our mobile, 24/7 societies (14, 41–43). Open hours and traffic flow can be direct influencing factors in accessibility and equity as time changes. This study found that the accessibility and equity of community healthcare during the daytime in the study area were generally higher, while they were lower during the night. Moreover, compared to traffic flow, the impact of opening hours on the equity of accessibility is more significant. These findings are potentially valuable for evaluating public facility locations and resource planning, yet they have been consistently overlooked in previous research (44, 45). Although traffic congestion remains a challenging social issue with no short-term solutions, the flexibility of hospital operating hours presents an opportunity. Improving community health services could be achieved through extended hospital hours or dynamic allocation of medical resources at varying times throughout the day.

The study also found that the transport modes are also important factors affecting accessibility and equity level. However, the predominant focus of accessibility equity studies in health research has been on the analysis of single transport modes, predominantly centered around car travel. As pointed by Tahmasbi et al. (46) and Hu et al. (47), the selection of transportation mode is related to multiple factors, including the patient’s health condition and income level, travel costs, traffic comfort, hospital’s strength, etc. In case of community hospitals in Nanjing, people typically visit for physical examinations, vaccines, or minor ailments, with riding and public transit being the most common modes of transportation for obtaining healthcare services. However, for other healthcare services such as hospital first aid, ambulances or private cars are frequently favored in the majority of cases due to time-saving considerations (48). Therefore, when assessing the spatial equity of public service facilities, the evaluation should be primarily based on the most predominant modes of transportation, rather than single mode or rarely selected modes of transportation.

Additionally, our results indicate that traditional static, rough methods of calculating travel time costs often lead to an overestimation of spatial accessibility or equity. This method was easy to implement in practice by utilizing GIS software, as it did not require consideration of many travel details and traffic dynamic characteristics, which was used extensively in previous study (49). However, the recent open data and novel data sources have made it possible to carry out more realistic and detailed spatial analyses (50, 51). To offer more reliable estimate, we distributed population data among grid cells instead of concentrating it at a single point in the administrative centre. Additionally, to obtain more precise OD travel time, we divided the real travel path into two parts: “on-road” path and “off-road” path. We utilized a network mapping approach to calculate “on-road” travel time, which has been demonstrated to be more straightforward, precise, and effective. In this study, the travel time of “off-road” path is not disregarded as previous studies (52, 53). Instead, it is quantified through pedestrian travel from an off-road point of origin or destination to the nearest road. This result is more objective, realistic, and reliable for depicting accessibility equity level.

## 5 Conclusion and limitation

This study is to investigate how the open hours of community hospitals, fluctuations in traffic flow, and different transport modes influence spatial accessibility and equity level. The conclusions obtained are as follows: (1) The proposed method combined the D2D model with the open OD API results is effective for accessibility computation of real transport modes. Our proposed approach is generic and widely applicable given that it is built on openly available input data sources that are available for hundreds of regions worldwide. (2) Spatial accessibility and equity of community healthcare experience significant fluctuations influenced by time variations. This insight could inform policymakers and urban planners in developing flexible strategies to address temporal variations in accessibility and equity, thereby enhancing the resilience and responsiveness of healthcare delivery systems. (3) The transportation mode is also a significant factor affecting accessibility and equity level. It is essential to not only consider optimizing the spatial and temporal distribution of community hospitals to enhance accessibility equity but also prioritize interventions that promote more equitable access to transportation options, especially for marginalized communities disproportionately affected by spatial disparities. These results are helpful to both planners and scholars that aim to build comprehensive spatial accessibility and equity models and optimize the location of public service facilities from the perspective of different temporal scales and a multi-mode transport system.

Our study has several limitations. Firstly, our study does not account for people’s spatiotemporal mobility. In fact, individuals from different social groups engage in complex activities at various times and locations. Therefore, there is a dynamic spatial distribution of the population over time in our societies. Future studies should aim to propose a comprehensive framework modeling spatial accessibility and equity that captures the dynamic nature of all three accessibility components (people, transport, activities) in dynamic cities. Secondly, due to constraints on obtaining OD travel time data at the same moment, the multilevel cell defining the study area was defined as 0.5 and 1 km. These limitations may reduce the accuracy of the study. Thirdly, while this study focuses on location-based accessibility and equity, this method pays more attention to the spatial relationship between geographical locations. However, there is a lack of consideration for individuals or special groups (such as older adult, office workers, students, etc.) who are active in geographical space. An important direction for future research is how to measure spatial accessibility equity for individuals or special groups engaged in various activities at different times and locations.

## Data availability statement

The raw data supporting the conclusions of this article will be made available by the authors, without undue reservation.

## Author contributions

JN: Conceptualization, Writing – original draft, Writing – review & editing. ZW: Data curation, Writing – review & editing. HL: Data curation, Writing – review & editing. JC: Data curation, Writing – original draft, Writing – review & editing. QL: Data curation, Visualization, Writing – review & editing.
